# The Impact of Exercise on Statin-Associated Skeletal Muscle Myopathy

**DOI:** 10.1371/journal.pone.0168065

**Published:** 2016-12-09

**Authors:** Hae R. Chung, Mayand Vakil, Michael Munroe, Alay Parikh, Benjamin M. Meador, Pei T. Wu, Jin H. Jeong, Jeffrey A. Woods, Kenneth R. Wilund, Marni D. Boppart

**Affiliations:** 1 Renal and Cardiovascular Disease Research Laboratory, Department of Kinesiology and Community Health, University of Illinois at Urbana-Champaign, Urbana, Illinois, United States of America; 2 Molecular Muscle Physiology Laboratory, Department of Kinesiology and Community Health, University of Illinois at Urbana-Champaign, Urbana, Illinois, United States of America; 3 Beckman Institute for Advanced Science and Technology, University of Illinois at Urbana-Champaign, Urbana, Illinois, United States of America; 4 Exercise Immunology Research Laboratory, Department of Kinesiology and Community Health, University of Illinois at Urbana-Champaign, Urbana, Illinois, United States of America; University of Louisville School of Medicine, UNITED STATES

## Abstract

HMG-CoA reductase inhibitors (statins) are the most effective pharmacological means of reducing cardiovascular disease risk. The most common side effect of statin use is skeletal muscle myopathy, which may be exacerbated by exercise. Hypercholesterolemia and training status are factors that are rarely considered in the progression of myopathy. The purpose of this study was to determine the extent to which acute and chronic exercise can influence statin-induced myopathy in hypercholesterolemic (ApoE^-/-^) mice. Mice either received daily injections of saline or simvastatin (20 mg/kg) while: 1) remaining sedentary (Sed), 2) engaging in daily exercise for two weeks (novel, Nov), or 3) engaging in daily exercise for two weeks after a brief period of training (accustomed, Acct) (2x3 design, n = 60). Cholesterol, activity, strength, and indices of myofiber damage and atrophy were assessed. Running wheel activity declined in both exercise groups receiving statins (statin x time interaction, p<0.05). Cholesterol, grip strength, and maximal isometric force were significantly lower in all groups following statin treatment (statin main effect, p<0.05). Mitochondrial content and myofiber size were increased and 4-HNE was decreased by exercise (statin x exercise interaction, p<0.05), and these beneficial effects were abrogated by statin treatment. Exercise (Acct and Nov) increased atrogin-1 mRNA in combination with statin treatment, yet enhanced fiber damage or atrophy was not observed. The results from this study suggest that exercise (Nov, Acct) does not exacerbate statin-induced myopathy in ApoE^-/-^ mice, yet statin treatment reduces activity in a manner that prevents muscle from mounting a beneficial adaptive response to training.

## Introduction

HMG-CoA reductase inhibitors (statins) reduce the synthesis of mevalonate, an important intermediary compound necessary for cholesterol production. Statins are the most effective pharmacological means of decreasing hypercholesterolemia and reducing cardiovascular disease (CVD) risk. They are considered a safe class of drugs, however, they carry a significantly elevated risk of skeletal muscle myopathy, ranging from fatigue and weakness to rhabdomyolysis, a potentially fatal condition. Myopathy is estimated to occur in 10% of all statin users [1, 2, 3, 4]. Recent results from the Statins on Skeletal Muscle Function and Performance (STOMP) clinical trial suggest no impact of long-term (6 months) atorvastatin on muscle strength or capacity, yet significant elevation of serum creatine kinase (CK) and myalgia [5, 6].

The biological basis for statin-induced myopathy is not well understood, yet *in vitro* and *in vivo* studies suggest that several factors may contribute, including apoptosis [7, 8, 9, 10], reduced isoprenoid and ubiquinone (coenzyme Q_10_, CoQ_10_) synthesis [11, 9, 12, 13, 14], and/or increased mitochondrial dysfunction [15, 16, 8, 12, 13, 17, 18]. *In vitro* and *in vivo* studies demonstrate that simvastatin can inhibit complex I [8, 17] and complex III [13] of the mitochondrial electron transport chain, which not only interferes with energy production, but also results in the generation of reactive oxygen species (ROS) that result in cellular damage. ROS can increase forkhead box (FoxO) transcription factor nuclear translocation [19] and subsequently upregulate atrogin-1 gene expression [20], an essential component of the ubiquitin proteasome pathway (UPP) and regulator of muscle protein degradation [21]. Statins can enhance atrogin-1 gene expression in rodents and human skeletal muscle, in a manner that may depend on FoxO dephosphorylation [22, 23]. Thus, mitochondrial dysfunction and ROS production and subsequent activation of the UPP via a FoxO-mediated signaling pathway may provide the stimulus for myofibrillar protein degradation, muscle weakness, and/or myalgia observed with HMG-CoA reductase inhibition.

Physical activity may increase the risk of statin-induced muscle myopathy and the prevalence of myopathy is estimated to be as high as 25% in individuals that engage in routine exercise while using statin medication [2, 24, 25]. Thompson *et al*. demonstrated that lovastatin can elevate serum CK following a single bout of eccentric exercise compared to lovastatin treatment alone [26]. In rodents, treadmill exercise can increase the severity of Type II fiber predominant muscle damage and mitochondrial degeneration associated with cerivastatin [27]. Genes associated with oxidative phosphorylation [28] and UPP [29] are significantly altered in human skeletal muscle as a result of statin administration in combination with eccentric exercise. Thus, engagement in exercise simultaneous with statin treatment appears to exacerbate myopathy, and mitochondrial dysfunction is likely responsible.

Repeated bouts of endurance exercise can stimulate mitochondrial biogenesis and improve respiratory capacity in a manner that is dependent on the transcriptional coactivator peroxisome proliferator-activated receptor gamma coactivator-1 alpha (PGC-1α) [30, 31]. Mitochondrial expansion provides a means to neutralize excessive oxidative stress [32]. In addition, PGC-1α can suppress FoxO3 activity and expression of FoxO target genes [33]. Together, these studies suggest the potential for exercise training to protect against statin-induced myopathy. Although there is some evidence to suggest that training *prior* to statin treatment can preserve mitochondrial function and muscle force in rodents [16, 34], the impact of repeated statin administration on maintenance of mitochondrial content and ROS production, as well as atrogin-1 mRNA and muscle damage has not been evaluated. In addition, hypercholesterolemia status is not generally accounted for in the majority of published rodent studies, a factor that can influence susceptibility to myopathy independent of statin use [35].

Mikus *et al*. [36] recently evaluated the impact of statins on beneficial adaptations associated with 12 weeks of supervised aerobic training in obese subjects at risk for metabolic disease, including maximal oxygen consumption (VO_2_ peak) and citrate synthase activity, a proxy marker for mitochondrial content. Whereas significant improvements were observed in control subjects, no beneficial changes were demonstrated in the simvastatin-plus-exercise group at the end of the study. Unfortunately, the extent to which intensity was maintained during the study was not reported, which is concerning given previous reports of reduced spontaneous physical activity with statin treatment [6]. Further information regarding the impact of statin treatment on training-mediated adaptations is warranted, especially given the current recommendation for exercise in the treatment of individuals at risk for CVD (frequent and prolonged bouts of mild to moderate intensity endurance training).

The purpose of this study was to determine the influence of exercise on statin-induced myopathy in a mouse model of hypercholesterolemia (ApoE^-/-^). We hypothesized that initiation of exercise training (novel exercise) at the onset of statin administration would exacerbate statin-induced myopathy, whereas exercise training initiated prior to statin administration would protect against statin-induced myopathy, in ApoE^-/-^ mice.

## Methods

### Study Overview

Protocols for animal use were approved by the Institutional Animal Care and Use Committee (IACUC) of the University of Illinois at Urbana-Champaign. To test the effects of simvastatin on muscle myopathy, 60 eight-week old, male apolipoprotein E-deficient mice (ApoE^-/-^) (Jackson Laboratory, Bar Harbor, ME) were randomized to six groups. In addition, identical experiments were conducted in 60 eight-week old male C57BL/6J mice (Jackson Laboratory) for comparison purposes. Mice were first assigned to one of three groups: sedentary (statin or saline injection in combination with sedentary conditions; Sedentary group), voluntary novel exercise (statin or saline injection in combination with exercise for two weeks; Novel group), or voluntary accustomed exercise (statin or saline injection in combination with exercise for two weeks following two weeks of exercise training; Accustomed group). Exercise was administered through the use of a running wheel. Mice were either injected with simvastatin (20 mg/kg/day) or an equivalent volume saline. The 12 groups (6 ApoE^-/-^ and 6 WT) are outlined in Table 1.

**Table 1:** Treatment groups based on combinations of cholesterol level, exercise, and statin treatment over days 1–14 and days 15–28.

Group	Day 1–14 of treatment	Day 15–28 of treatment
WT/Saline/Sed	Sedentary	Sedentary, Saline injection
WT/Statin/Sed	Sedentary	Sedentary, Statin injection
ApoE/Saline/Sed	Sedentary	Sedentary, Saline injection
ApoE/Statin/Sed	Sedentary	Sedentary, Statin injection
WT/Saline/Novel	Sedentary	Running wheel, Saline injection
WT/Statin/Novel	Sedentary	Running wheel, Statin injection
ApoE/Saline/Novel	Sedentary	Running wheel, Saline injection
ApoE/Statin/Novel	Sedentary	Running wheel, Statin injection
WT/Saline/Accustomed	Running Wheel	Running wheel, Saline injection
WT/Statin/Accustomed	Running Wheel	Running wheel, Statin injection
ApoE/Saline/Accustomed	Running Wheel	Running wheel, Saline injection
ApoE/Statin/Accustomed	Running Wheel	Running wheel, Statin injection

WT = C57BL/6J wild type mice; ApoE = apolipoprotein E-deficient (ApoE^-/-^) mice.

### Running Wheel Exercise

Mice were monitored over a period of 28 days, housed in separate cages, fed a standard chow diet and provided water *ad libitum*, and kept on a 12/12 hour light/dark cycle. Mice assigned to the accustomed exercise group were provided access to an 11.5 cm running wheel (Mini-Mitter, Bend, Oregon) in their cage on day one, while novel exercise mice were not given a running wheel until day 15. Activity levels were recorded until the 28^th^ day. Distance was monitored using magnetic reed switches (Mini Mitter) and a bicycle computer (Sigma). Activity was recorded every 24 hours at the time medication was administered.

### Statin Injections

Starting on day 15 and continuing until day 28, each mouse received an intraperitoneal injection of saline or statin every 24 hours. The majority of mice run during the dark cycle, so injections were given towards the start of the light cycle to minimize any direct effects of the injections [34]. Simvastatin/Zocor (Cat # 4893, Medical Isotopes Inc.) was given at a dose of 20 mg/kg/day from a solution of 0.25 mg/ml in sterile saline with a sterile insulin needle [37]. Accounting for allometric species differences, this dosage translates to a Human Equivalent Dosing of 1.6 mg/kg/day according to guidelines provided by the 2005 FDA Center for Drug Evaluation and Research. This dose was chosen due to pilot research conducted by this group, which demonstrated that 1 mg/kg and 10 mg/kg doses of simvastatin were too small to show any considerable change in muscle force in only 2 weeks.

### Grip Strength Test

Three days prior to the start of the running wheel exercise for the accustomed group (day -2) all mice had their all-limb grip strength tested using a force gauge (Columbus Instruments, Columbus, OH). The mice were held up to the horizontal grip and then were pulled steadily backwards until they could not hold on any longer. This was repeated 3 times per mouse for each grip, and the highest force measurement was recorded. This procedure was repeated 2 days prior to the start of the exercise (day -1), and the highest number from both days was recorded as their maximum strength. This test was then administered to all mice on days 13, 20, and 27 of the study.

### Isometric Force Measurement

Hindlimb plantarflexor isometric force testing was conducted in all mice on the 29^th^ day of the study, approximately 24 hours following final injection. Mice were anesthetized with ketamine and xylazine, then the left sciatic nerve was dissected through the thigh. Electrodes were attached to the nerve and stimulated at 250 Hz for 1.5s to provoke a maximum force plantarflexor contraction. Strength was measured using a plate attached to a servomotor (305C-LR; Aurora Scientific). The limb was stimulated 10 times with a time interval of 5s between each stimulation to assess fatigability. As a measure of fatigue, the tenth contraction was compared with the maximum contraction and expressed as a percentage of maximal force.

### Blood Draws and Tissue Collection

One day prior to the start of the running wheel exercise for the accustomed group (day 0) all the mice had approximately 0.3 mL of blood drawn via their jugular vein using an animal lancet (Bioseb) stored in an ethylene-diamine tetra-acetic acid (EDTA)-treated tube (Bioseb). Blood was again collected using this method on day 14. On day 29, after isometric force testing and euthanasia, approximately 1 mL of blood was extracted from each mouse via their inferior vena cava using heparin-treated syringes and collection tubes. The samples were centrifuged at 1200g for 10 minutes at 4°C, and plasma was collected and stored at -80°C. Gastrocnemius muscles were also collected and stored at -80°C.

### Blood Chemistry

Plasma total cholesterol was measured by standard enzymatic methods using commercially available assay kits (Infinity Incorporated, Melbourne, Australia). Plasma 4-hydroxynonenal (4-HNE), a marker of oxidative stress, was measured in duplicate using a commercially available ELISA kit (Cell Biolabs OxiSelect^™^ HNE Adduct ELISA kit, San Diego, CA).

### RNA and DNA Extraction

Mice were euthanized by cervical dislocation under anesthesia and gastrocnemius muscle was dissected. RNA was extracted from 50–100 mg of gastrocnemius muscles tissue using Trizol reagent (Invitrogen, Carlsbad, CA) according to manufacturer’s instructions, and then quantified using spectrophotometry. After the separation of RNA and protein from the tissue, DNA was extracted with phenol-chloroform then precipitated with ethanol, according to manufacturer’s instructions. Spectrophotometry was used to quantify the amount of RNA and DNA extracted.

### Evaluation of Gene Expression

Real-time RT-PCR for the genes of interest was conducted using the following procedure: one cycle at 48°C for 30 minutes, followed by 95°C for 10 minutes, then 40 cycles of 95°C for 15 seconds and 60°C for 1 minute. This procedure was conducted using an ABI PRISM 7700 sequence detector (Applied Biosystems, Roche, Branchburg, NJ) and a Taqman 100R × n PCR Core Reagent Kit (Applied Biosystems). Primers (PGC-1α, Cat # 4331182 and atrogin-1, Cat # 4310893E; Applied Biosystems) were recreated using Primer Express Software version 2.0 (Applied Biosystems). The data was normalized by dividing the target amount by the amount of glyceraldehyde-3-phosphate dehydrogenase (GAPDH), which served as an internal control and was used as the housekeeping gene, and all data are presented relative to its expression using the ΔΔCt method.

### Real-Time PCR for Mitochondrial DNA

The ratio of mitochondrial DNA to nuclear DNA provides an assessment of the volume of mitochondria per cell in a given tissue. Nuclear DNA and mtDNA was amplified and quantified using real-time PCR with an ABI PRISM 7700 sequence detector (Applied Biosystems). A 120-bp long region of mtDNA was amplified for quantification by PCR and cloned into the plasmid pCDNAII, following the manufacture’s procedure (Invitrogen), then sequenced to verify the identity of the DNA. The DNA concentration was estimated by spectrophotometry and calculated to give a stock of 2.5E10 copy/μL. Amplification and quantification prior to cloning was completed using the following PCR procedure: one cycle at 50°C for 2 minutes, followed by 95°C for 10 minutes, then 40 cycles of 95°C for 15 seconds and 60°C for 1 minute. The amount of DNA was quantitated using the following assay during PCR: 50 μL containing 10 μL DNA template, 11 μL of 25 mmol/L MgCl_2_, 0.05 μL AMPErase UNG (uracil-N-glycosylase), 15.25 μL DI water, 0.25 μLAmpliTaq Gold DNA Polmerase, 1 μL of each dNTP, and 5 μL of 10X buffer A. The copy numbers of the unknown was determined by creating a standard curve from a plasmid of known copy number. These results were normalized by also amplifying the 120-bp region, then cloning it (as described above).

### Immunohistochemistry

Gastrocnemius cross sections were taken at the mid belly and fixed in acetone (-20°C) for 10 min. For assessment of myofiber damage, sections were stained with FITC-conjugated anti-mouse IgG antibodies (FI2000) (Vector Laboratories, Burlingame, CA). To assess centrally located nuclei (CLN) content, sections were stained with rabbit anti-mouse dystrophin (Abcam, Cambridge, MA) primary antibody and 4’,6-diamidino-2-phenylindole (DAPI). For assessment of myofiber CSA, sections were co-stained with either mouse IgG2b anti-myosin heavy chain 1 (BA-D5) and mouse IgM anti-myosin heavy chain 2X (6H1) or co-stained with mouse IgG1 anti-myosin heavy chain 2A (Sc-71) and mouse IgM anti-myosin heavy chain 2B (BF-F3). All primary antibodies were obtained from the Developmental Studies Hybridoma Bank (Iowa City, IA, USA). Individual fibers were outlined with rabbit anti-mouse dystrophin (Abcam) primary antibody. Secondary antibodies included Alexa Fluor 350 conjugated anti-mouse IgG2b (Invitrogen), AMCA conjugated anti-mouse IgM μ chain specific (Jackson Immunoresearch, West Grove, PA), Alexa Fluor 488 conjugated anti-mouse IgG subclass 1 (Jackson Immunoresearch), and Alexa Fluor 633 goat anti-rabbit (Invitrogen). Images were captured at 10x magnification using a Leica DMRXA2 microscope and Axiovision software (Zeiss, Thornwood, NY). To assess muscle damage, IgG^+^ fibers were counted from three sections per sample, and the total number of IgG^+^ fibers was normalized to the total area analyzed (mm^2^). For CLN assessments, the total number of myofibers and fibers containing CLN for each sample were counted using ImageJ (NIH, Bethesda, MD). To assess myofiber CSA, Adobe Photoshop was used to quantitate images acquired with a Zeiss AxioCam digital camera. Briefly, co-stained images of dystrophin and type-specific fiber types were acquired at 10x magnification from each sample, then imported into Adobe Photoshop (CS5 Extended) where up to 400 fibers per sample were manually circled using the magnetic lasso tool, which grabs the positively stained pixels and decreases subjectivity and interassessment error. The CSA for each fiber was recorded in a measurement log. The results for each sample were then averaged.

### Statistical Analysis

All outcome measures were separately analyzed and reported for ApoE^-/-^ mice. Continuous variables are presented as means ± standard deviation (SD). Outcomes at baseline were tested for difference between groups by one-way analysis of variance (ANOVA). The effects of drug treatment (saline, statin) and activity (sedentary, novel, accustomed) on changes in variables related to muscle myopathy were tested by two-way ANOVA when no difference between groups was found at baseline. Paired t-tests were conducted to examine within-groups difference (saline vs statin) at the end of the experiment. Additionally, repeated measures ANOVA was used to test the effects of drug and exercise on serial changes in grip strength, daily running wheel activity, and body weight during the course of the 4-week study. Strength was normalized to body weight only when differences in body weight were detected between groups. Tukey or LSD post-hoc analysis was performed to examine group differences only if a significant interaction was detected for variables. All statistical analyses were performed using SPSS version 19 and 22 (IBM, Chicago, IL) and statistical significance was defined as p<0.05.

## Results

### Body and Muscle Weight

No significant differences in body weight were detected between groups for either ApoE^-/-^ or WT mice. However, final body weights were significantly increased in both ApoE^-/-^ and WT mice compared to initial body weights, in accordance with expected natural growth rates over the duration of the experiment (p<0.001 for both genotypes) (Table 2). In addition, muscle weight was not significantly different between groups for either ApoE-/- or WT mice throughout the study (data not shown).

**Table 2:** Body Weight.

	Wild Type		ApoE^-/-^	
Group	Initial BW (g)	Final BW (g)*	Initial BW (g)	Final BW (g)*
Sed/Saline	21.89 ± 1.62	23.42 ± 1.25	23.40 ± 1.58	25.17 ± 1.31
Sed/Statin	22.05 ± 1.35	22.70 ± 2.95	22.66 ± 3.10	24.58 ± 1.65
Novel/Saline	22.76 ± 2.41	23.59 ± 1.71	23.45 ± 3.62	25.29 ± 2.15
Novel/Statin	22.11 ± 1.79	23.96 ± 1.72	22.77 ± 3.15	24.97 ± 1.17
Accustomed/Saline	22.56 ± 1.36	23.38 ± 0.63	23.63 ± 2.47	24.99 ± 1.96
Accustomed/Statin	22.06 ± 1.40	23.19 ± 1.49	21.13 ± 1.26	23.77 ± 1.34

Data expressed as mean ± SD,

* p<0.001 compared with initial body weight (BW), n = 8-10/group

### Cholesterol

**Fig 1:**
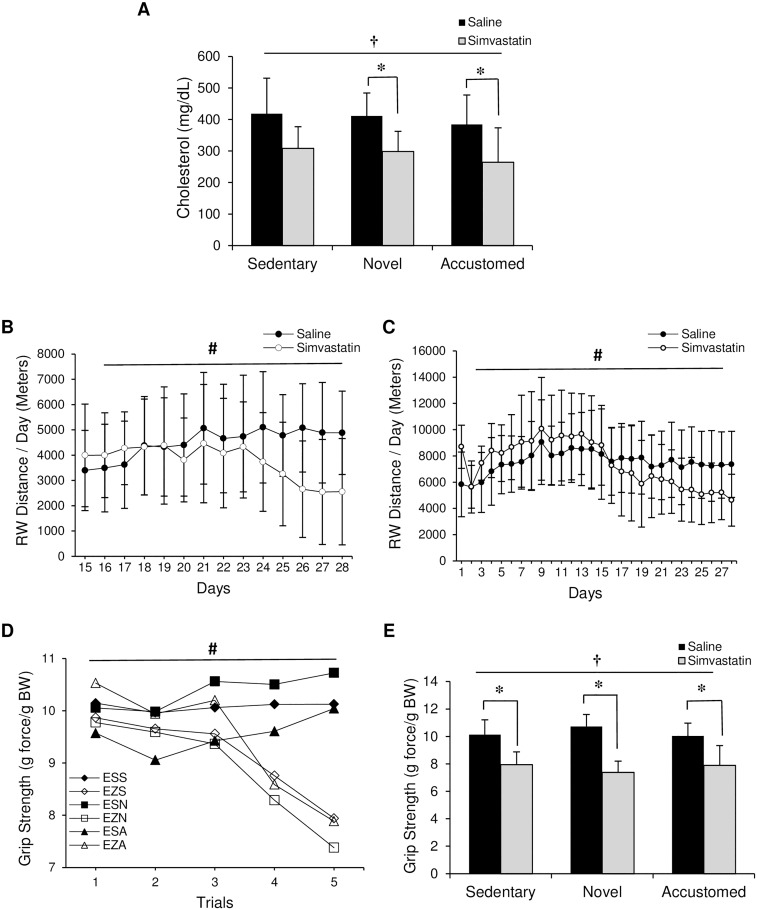
Effect of statin treatment and exercise on cholesterol, voluntary running wheel activity, and maximal grip strength. ApoE^-/-^ mice were evaluated following normal cage activity (Sedentary), two weeks of wheel running in combination with saline or simvastatin (20 mg/kg) (Novel), or two weeks of wheel running in combination with saline or simvastatin after two weeks of exercise training (Accustomed). (A) Cholesterol level at the end of the intervention, (B) running wheel distance for novel exercise group, (C) running wheel distance for accustomed group, (D) grip strength, and (E) change in grip strength from beginning to end of study. E: ApoE^-/-^, Z: Zocor (simvastatin), S: Saline, S: Sedentary, N: Novel, A: Accustomed. Means ± SD; n = 8-10/group. † = main effect of statin, p<0.05; ^#^ = statin x time interaction, p<0.05; * = within group pairwise comparison, p<0.05.

There was no difference in plasma cholesterol levels between groups at baseline. Statin treatment lowered cholesterol in ApoE^-/-^ mice (statin main effect, F(1,43) = 17.68, p<0.001) (Fig 1A). Plasma cholesterol was significantly reduced in both novel and accustomed exercise groups receiving statin treatment compared to respective saline controls (p<0.001). In WT mice, statin treatment had no effect on cholesterol level (data not shown). Results for running wheel activity, muscle function, and adaptation were nearly identical between ApoE^-/-^ and WT mice throughout the study. Therefore, only the results for ApoE^-/-^ mice are presented.

### Running Wheel Activity

Daily running wheel distances for 14 days (Day 15–28) in the novel and 28 days in the accustomed exercise groups are shown in Fig 1B and 1C, respectively. In both novel and accustomed exercise groups, there was a statin x time interaction, with decreasing activity over the course of the study in the statin groups (p<0.05).

### Maximal Grip Strength

There was no difference between groups at baseline. However, over the course of the study, all-limb grip strength in ApoE^-/-^ mice showed a significant statin x time interaction effect for maximal relative strength (p<0.001) (Fig 1D). Further analysis comparing the differences at each measured time point revealed that voluntary maximal strength was significantly lower with statin treatment in all groups compared to saline at week 3 (Trial 4) and week 4 (Trial 5) (p<0.001 for all). Voluntary maximal grip strength normalized to body weight at the end of the experiment showed a significant effect of statin treatment (statin main effect, F(1,53) = 106.732, p<0.001). Maximal grip strength was lower in the statin groups compared saline-treated controls based on paired t-tests (F(1,53) = 106.73, p<0.001 for all 3 groups) (Fig 1E).

### Maximal Isometric Muscle Force

**Fig 2:**
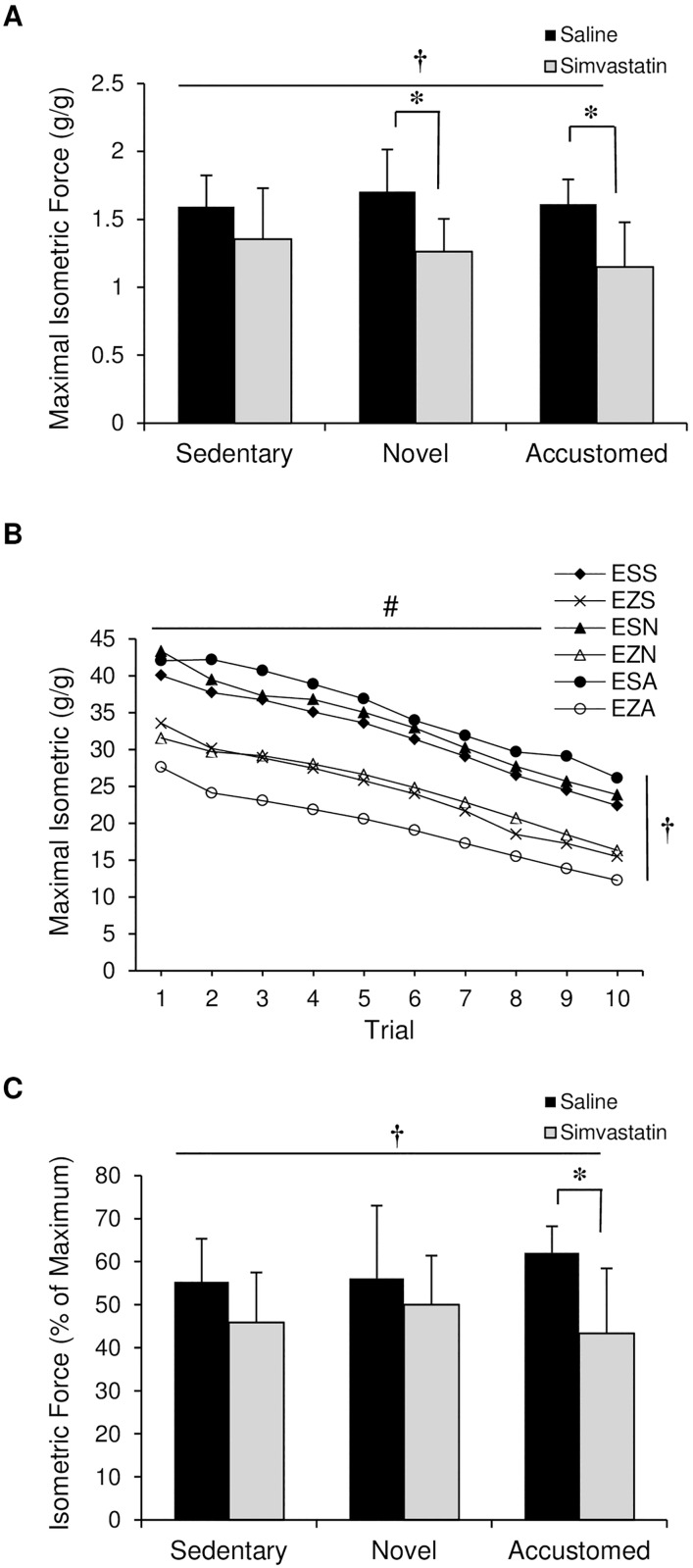
Effect of statin treatment and exercise on maximal isometric force. The sciatic nerve of ApoE^-/-^ mice was stimulated and hindlimb isometric force was evaluated following normal cage activity (Sedentary), two weeks of wheel running in combination with saline or simvastatin (20 mg/kg) (Novel), or two weeks of wheel running in combination with saline or simvastatin after two weeks of exercise training (Accustomed). (A) Maximal isometric force, (B) maximal isometric force produced with repeated stimulation to provide a measure of fatigability, (C) difference in force between initial stimulation and after 10 events to provide a measure of fatigability. E: ApoE^-/-^, Z: Zocor (simvastatin), S: Saline, S: Sedentary, N: Novel, A: Accustomed. Means ± SD; n = 8-10/group. † = main effect of statin, p<0.05; ^#^ = time main effect, p<0.05; * = within group pairwise comparison, p<0.05.

A significant main effect for statin treatment was found on maximal isometric force as assessed by sciatic nerve stimulation at the end of the experiment (statin main effect, F(1,48) = 22.322, p<0.001) (Fig 2A). Post-hoc revealed significant group differences only in the novel and accustomed exercise groups (p<0.001).

### Muscle Fatigue (% of Maximum Isometric Force)

Isometric force with repeated stimuli was reduced over time in all groups (time main effect by repeated measures ANOVA, p<0.001) (Fig 2B). Statin treatment increased susceptibility to muscle fatigue (statin main effect, F(1,48) = 10.654, p<0.002) (Fig 2C). The extent of force loss was higher following statin treatment with accustomed exercise compared to the respective saline group (p<0.002) (Fig 2C).

### PGC-1α mRNA

**Fig 3:**
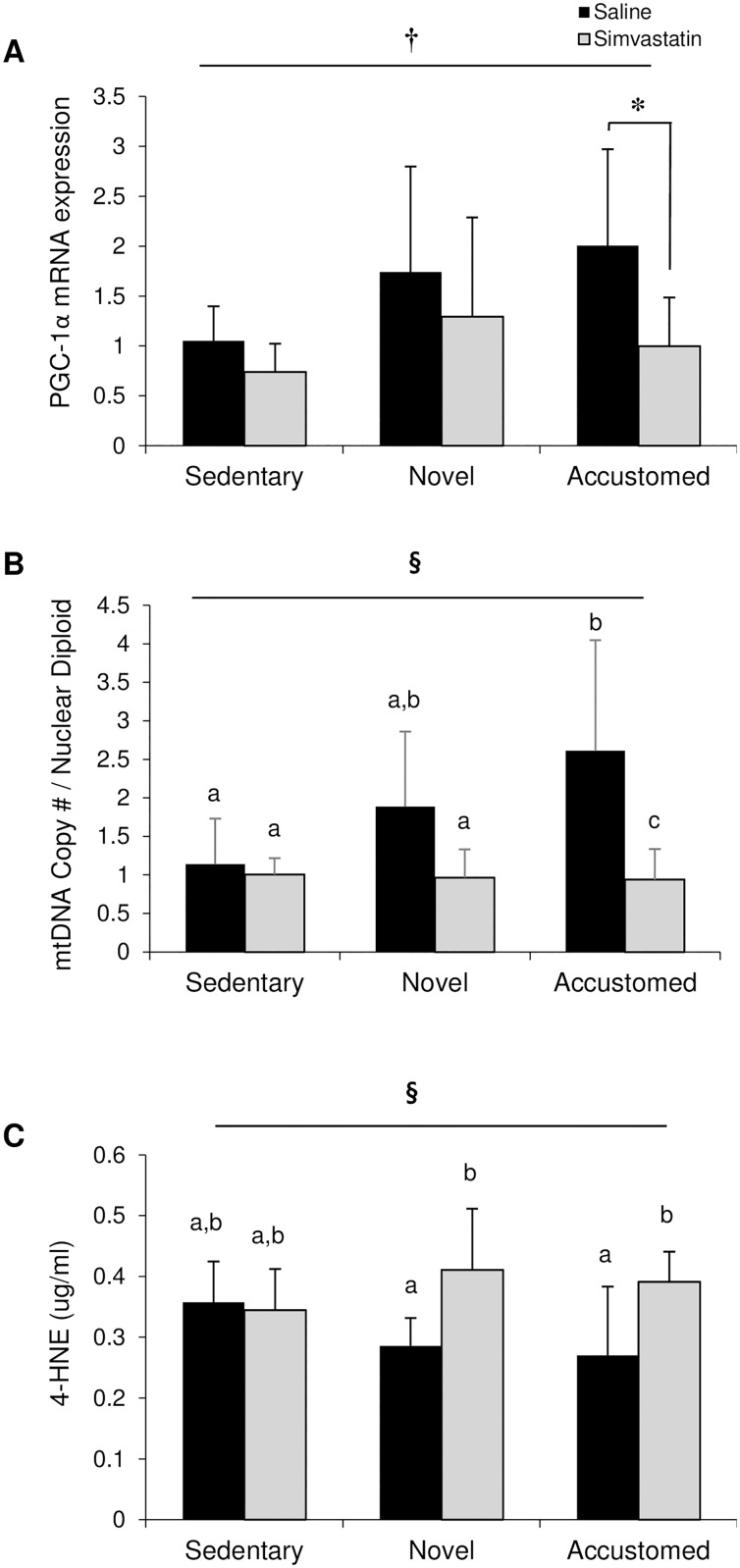
Statin treatment prevents beneficial adaptations obtained following accustomed exercise. (A) PGC-1α mRNA and (B) mitochondrial DNA content in gastrocnemius muscles, and (C) plasma 4-HNE after normal cage activity (Sedentary), two weeks of wheel running in combination with saline or simvastatin (20 mg/kg) (Novel), or two weeks of wheel running in combination with saline or simvastatin after two weeks of exercise training (Accustomed). Means ± SD; n = 8-10/group. † = main effect of statin p<0.05; ^**§**^ = statin x exercise interaction, p<0.05; ^a,b,c^ = different letters indicate statistically significant differences at 95% confidence.

PGC-1α mRNA, mitochondrial content, and 4-HNE from excised gastrocnemius muscles were examined to provide an assessment of training status post-exercise in the presence and absence of statin treatment. Skeletal muscle PGC-1α mRNA expression was lower in mice receiving statin treatment compared to mice that received saline (statin main effect, F(1,34) = 6.053, p = 0.019) (Fig 3A). PGC-1α mRNA expression was significantly lower following statin treatment with accustomed exercise compared to similarly exercised mice that received saline treatment (p = 0.018).

### Mitochondrial DNA Content (mtDNA)

Mitochondrial DNA content in skeletal muscle was altered by statin treatment and exercise (statin x exercise interaction, F(2, 42) = 4.632, p = 0.015) (Fig 3B). The accustomed group had an increased mitochondrial DNA content concurrent with saline injection compared to the sedentary control group and all mice receiving statin treatment (p<0.001).

### 4-Hydroxynonenal (4-HNE)

Plasma 4-HNE was altered with statin and exercise intervention (statin x exercise interaction, F(2,35) = 3.555, p = 0.039) (Fig 3C). 4-HNE levels were higher in the novel (p = 0.006) and accustomed (p = 0.006) exercise groups compared to saline-treated controls.

### Serum Amyloid A

**Fig 4:**
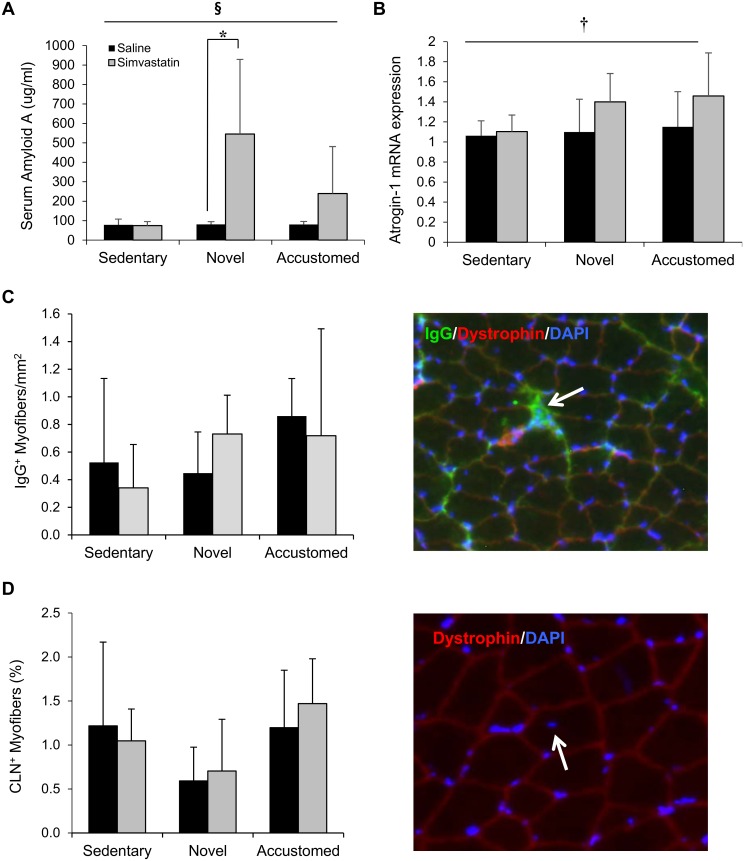
Statin treatment in combination with novel or accustomed exercise increases serum amyloid A content and atrogin-1 gene expression in skeletal muscle, but does not increase fiber damage or impact fiber repair. (A) Serum amyloid A content and (B) atrogin-1 mRNA, (C) IgG^+^ myofibers, and (D) CLN^+^ myofibers in gastrocnemius muscles after normal cage activity (Sedentary), two weeks of wheel running in combination with saline or simvastatin (20 mg/kg) (Novel), or two weeks of wheel running in combination with saline or simvastatin after two weeks of exercise training (Accustomed). Representative images of IgG^+^ and CLN^+^ fibers in muscle are presented for (C) and (D). Means ± SD; n = 8-10/group. ^**§**^ = statin x exercise interaction, p<0.05; † = main effect of statin, p<0.05; * = within group pairwise comparison, p<0.05.

Serum amyloid A, a proxy marker for systemic inflammation, was altered by statin treatment and exercise (statin x exercise interaction, F(2, 33) = 5.334, p = 0.010) (Fig 4A). Serum amyloid A levels were significantly higher in the novel exercise statin group compared to saline treatment (p = 0.009). After combining novel and accustomed exercise groups, a statin x exercise interaction remained (p = 0.025) and statin treatment increased serum amyloid A in response to exercise compared to saline (p = 0.001).

### Atrogin-1

Atrogin-1 mRNA was higher in statin-treated mice compared to saline controls (statin main effect, F(1,35) = 4.381, p = 0.044) (Fig 4B). After combining novel and accustomed exercise groups to increase the power to detect the effects of exercise, there was a significant interaction effect of statin × exercise (p = 0.023), with an increase in atrogin-1 mRNA in the statin and combined exercise group (combined group data not shown).

### Muscle Fiber Damage and Repair

Muscle fiber damage and repair (as assessed by IgG staining and CLN^+^ fiber counting) was minimally observed in any sample. No differences in the number of IgG^+^ fibers per area of tissue were detected between groups (Fig 4C). The percentage of fibers with a CLN was not different among groups, yet a trend toward a decrease with novel exercise was observed (exercise main effect, F(2,24) = 3.343, p = 0.05) (Fig 4D).

### Muscle Fiber CSA

**Fig 5:**
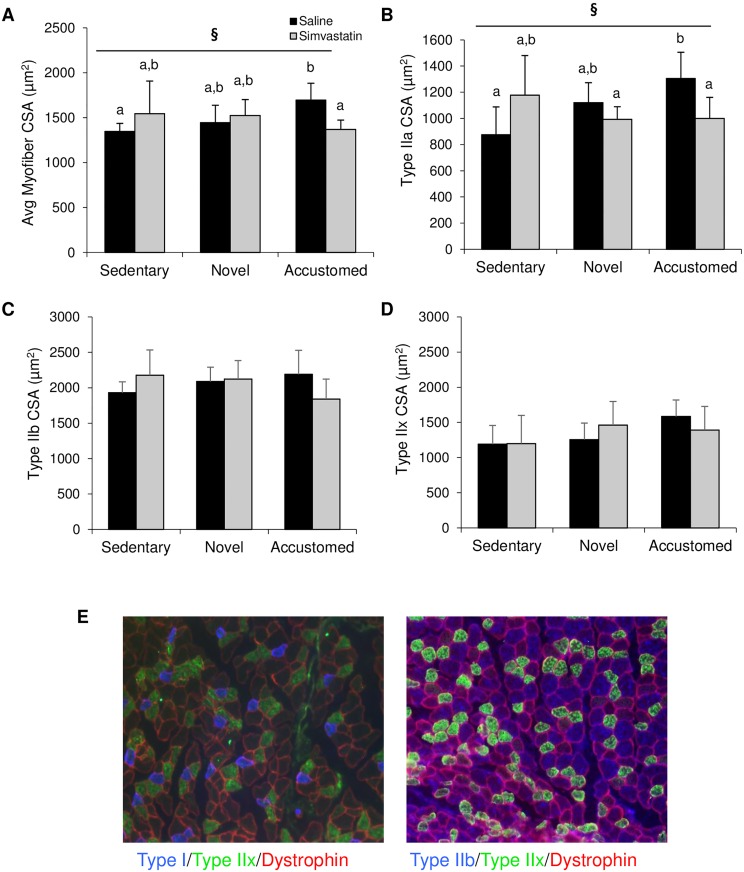
Statin treatment does not significantly impact fiber type specific cross sectional area. (A) Average myofiber CSA (all types combined), (B) representative image of CSA staining, (C) average Type 2A CSA, (D) average Type 2B CSA, and (E) average Type 2X CSA after normal cage activity (Sedentary), two weeks of wheel running in combination with saline or simvastatin (20 mg/kg) (Novel), or two weeks of wheel running in combination with saline or simvastatin after two weeks of exercise training (Accustomed). Representative images of fiber types in muscle are presented for (E). Means ± SD; n = 4-6/group. ^**§**^ = statin x exercise interaction, p<0.05; ^a,b^ = different letters indicate statistically significant differences at 95% confidence.

The average CSA of all analyzed myofibers (independent of fiber type) was altered with statin treatment and exercise (statin x exercise interaction, F(2,24) = 4.176, p = 0.03) (Fig 5A). CSA was increased in the accustomed group with saline injection compared to the sedentary, saline control group and the increase was abrogated by statin injection (Fig 5A). Type IIA CSA was altered by statin treatment and exercise (statin x exercise interaction, F(2,24) = 6.053, p = 0.01) (Fig 5B). Type IIA CSA was similarly increased in the accustomed group with saline injection compared to the sedentary, saline control group and the increase was abrogated by statin injection (Fig 5B). The CSA of both Type IIB and IIX fibers were not significantly different between groups (Fig 5C and 5D). Neither statin treatment nor exercise affected fiber type composition (total % of fiber types) (data not shown).

## Discussion

The purpose of this study was to examine the influence of exercise on simvastatin-induced myopathy in hypercholesterolemic (ApoE^-/-^) mice. We found that two weeks of statin treatment significantly impaired muscle function as demonstrated by decreased running wheel activity, grip strength, and maximal isometric force (main effect of statin treatment, p<0.05). Exercise training increased mitochondrial content and myofiber CSA while decreasing oxidative stress (4-HNE) in mice receiving saline, yet these beneficial adaptations were compromised concomitant with statin treatment. Statin treatment increased atrogin-1 mRNA, particularly in both exercise groups, yet muscle damage and atrophy were not noted in any group. Overall, the results from this study suggest that exercise does not exacerbate statin-induced myopathy compared to sedentary conditions, nor does training protect against statin-induced myopathy, in hypercholesterolemic mice.

The present study demonstrated that two weeks of statin treatment can significantly impair muscle function, as evidenced by decreased running wheel activity (novel and accustomed exercise groups), voluntary grip strength, and maximal isometric force, as well as increased fatigue (Figs 1 and 2). Training prior to statin treatment did not preserve function, and reductions were similar between all groups. Recent results from the STOMP clinical trial suggest no significant impact of atorvastatin on muscle strength or capacity, but rather increased incidence of myalgia [6]. However, declines in muscle strength have been previously reported during the course of statin therapy [38, 39]. It is interesting to note that *post hoc* analyses only detected a significant decrease in maximal isometric force with statin treatment in both exercise groups compared to respective saline control groups. Similarly, serum Amyloid A and atrogin-1 mRNA were not altered with statin treatment under sedentary conditions, yet elevated with exercise (Figs 3C and 4). Although speculative, these data suggest that exercise may have increased susceptibility to myopathy to some extent, but the full impact of combined treatment was not evident during the short period of training.

Meador *et al*. evaluated the impact of training (accustomed exercise) on statin-induced myopathy compared to initiation of exercise concurrent with statin therapy (novel exercise) [34]. Accustomed, but not novel exercise, preserved muscle function as measured by maximal isometric force. This result is in direct contrast to our findings, which suggest no protection. We cannot account for the difference between studies other than the source of statin (cerivastatin in the earlier study and simvastatin in the current). In the current study, daily wheel running distances gradually decreased after statin treatment started, a response not observed with saline treatment. The fact that activity level and grip strength were *gradually*, not rapidly, reduced may account for the discrepancy between our results and a separate *in vitro* study conducted by Bouitbir *et al*. [16]. In that study, investigators evaluated the impact of atorvastatin on mitochondrial function (V_max_ and H_2_O_2_) in permeabilized fibers dissected from muscle post-training. Training indeed preserved mitochondrial function, yet fibers were only acutely exposed to statin treatment (single application). Thus, we speculate that protection provided by training may not be sustained upon repeated statin exposure.

Exercise training increases mitochondrial content and improves mitochondrial function in a manner that is dependent on PGC-1α transcription [30, 31]. The exercise-induced increase in mitochondrial content has been shown to effectively neutralize excessive oxidative stress [32]. In the present study, mitochondrial content was enhanced and 4-HNE was decreased in saline-treated exercise groups compared to sedentary mice. In addition, Type IIA myofiber CSA was also enhanced post-training. Unfortunately, these beneficial adaptations were abrogated in statin-treated mice, perhaps due in part to the decline in activity. Whether a longer period of training (>2 weeks) would have provided better protection is not known, but doubtful given the evidence of successful adaptation in the present study.

Hypercholesterolemia can increase susceptibility to muscle damage [35], delay healing [40], and negatively impact muscle function [41]. Thus, we originally hypothesized that cholesterol status may independently influence statin-induced myopathy. However, in the present study, we did not observe any difference in myopathy or adaptation between ApoE^-/-^ and WT mice in response to statin treatment, with or without exercise. Therefore, it appears that the detrimental effects of statins on muscle damage are not confounded by cholesterol level in a murine model.

Overall, the results from this study suggest short-term exercise does not significantly exacerbate statin-induced myopathy, as strength was reduced to the same extent in all groups upon statin administration. Enhancement of systemic inflammation and atrogin-1 mRNA in the exercise groups compared to sedentary suggest some potential for myopathy to worsen with time. Thus, long-term follow up studies should be conducted. Physicians traditionally advocate a combination of statin treatment with a wellness program that includes exercise. However, the results from this study and others suggest that statin treatment may inhibit physical activity, and lack of engagement in activity may prevent the opportunity to reap additional benefits, such as weight maintenance and preservation of cognition and mental health. Clearly, the administration of statin medication should be advocated only when necessary and additional preclinical studies are warranted to understand the mechanistic basis for the decline in activity with statin treatment.
